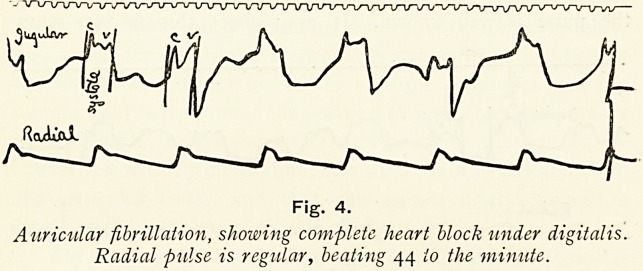# Auricular Fibrillation
1A paper read before the Bristol Dispensary Clinical Society.


**Published:** 1911-06

**Authors:** C. E. K. Herapath


### AURICULAR FIBRILLATION.[1]

BY

C. E. K. HERAPATH, M.D. Lond.

THE subject of this paper is a condition of irregularity of the heart which is quite commonly met with in clinical practice. Much work has been done on the subject recently both in the recognition and classification of the condition, and in the way these hearts react to drugs. I hope that what I have written may help in the recognition of the condition as a clinical entity, and perhaps in a small way also towards its treatment.

Auricular fibrillation is characterised by a persistently irregular pulse, both of force and rhythm, with usually more or less severe heart failure. It is met with slightly more frequently in women than men, and occurs generally in middle life, though cases in children, young adults, and old people are not rare. It is found chiefly in two heart conditions, (1) in the later stages of rheumatic mitral stenosis, and (2) in elderly

[1] A paper read before the Bristol Dispensary Clinical Society.

people with arterio-sclerosis and fibrous degeneration of the cardiac wall. In the last nine months I have taken tracings with Mackenzie's Polygraph from twenty-six cases of irregular heart, and of these fourteen were found to have fibrillation of the auricles, nine of which had a history of rheumatism. Of the fourteen, eight were women and six men. The condition is easily recognised by taking simultaneous tracings from the radial and jugular pulses (Fig. 1). The jugular pulse obtained from the neck should consist of at least three waves, one of which, the carotid or "c" wave, is found by measurement from the radial—it occurs $\frac{1}{10}$ sec. before the radial upstroke. The systole of the auricle, which takes place $\frac{1}{5}$ sec. before that of the ventricle, should give a wave—the "a" wave—$\frac{1}{5}$ sec. before

Fig. 1.

*Normal tracing, showing the diastolic "a" wave and the systolic waves. In all tracings the time marker shows $\frac{1}{5}$ sec.*

the "c" wave. The third or ventricular wave is not of importance to us to-night, save that it occurs during ventricular systole. Thus there are two waves during ventricular systole and one during diastole. The diastolic or "a" wave is absent in cases of auricular fibrillation, so that all waves occur during systole of the ventricle; and for this reason this type of pulse is called the ventricular type, in contradistinction to the normal or auricular type, where the "a" wave is present. On examining the radial pulse, one is struck by its absolute irregularity. Beats of all sizes are indiscriminately following one another, and the size of the wave does not at all seem to depend on the length of the pause preceding it, as is usually the case. Large beats are found after a short pause, and vice versa. If the length of the successive beats be accurately measured, it is found that beats of the same length practically never come together. This condition of pulse has earned for itself the name of pulsus irregularis perpetuus and delirium cordis. On auscultation of such a heart, the sounds are extremely difficult to analyse, for they come so rapidly and irregularly that it is often impossible to say which are first and which second sounds. Often there is a series of rapid beats, which may be followed either by a few slow ones or by another series of rapid ones. There may be murmurs—in the rheumatic and mitral stenotic cases there are almost always a systolic or a loud diastolic murmur—provided the heart be not beating too rapidly, when often none can be discovered. An important point is that a crescendo murmur is not heard in these cases. The reason for this I will

Fig. 2.

*Tracing of auricular fibrillation, showing irregularity of radial and the ventricular form of venous pulse. There is no "a" wave preceding the "c" wave.*

state later. On inspection of the veins in the neck, one sees a prominent pulsation in them which is systolic in time, and there is usually much dilatation and engorgement of the venous system.

These cases were first classified by James Mackenzie, who suggested that auricle and ventricle were contracting simultaneously, owing to the stimulus arising in the auriculo-ventricular node of Tawara, instead of in the normal situation at the root of the great veins. He thus accounted for the absence of the "a" wave in the jugular tracing, and called the condition "nodal rhythm." Since this much work has been done both in Germany and in England, the chief English worker being Thomas Lewis, of University College, London. He published the result of his work in the journal *Heart*, March, 1910, proving that in this condition the auricles were not contracting properly, but were in a state of fibrillation somewhat similar to that which occurs in the muscles in progressive muscular atrophy. His methods were as follows. He produced fibrillation of the auricle in dogs by means of electrical stimulation to the exposed heart, and while in this state he noticed that the ventricles took on this disorderly rhythm. On taking electro-cardiograms, a method by which the electrical variations of the heart-muscle contractions are registered by photographs of the movements of a string galvanometer, he found that they were identical with those taken from human beings with this kind of pulse, and further he has on two occasions seen the heart of a horse afflicted with this disease beating in situ.

We may therefore, I think, take it as proven that the auricles do not contract normally, but are in a continual state of fibrillation. This explains why a presystolic murmur does not occur; for in spite of criticism, there is not much doubt that a presystolic murmur is the result of auricular contraction.

Now let us consider how this state of things affects the heart. Normally, ventricular contraction is caused by stimuli arising from the roots of the great veins, passing through the auricular wall, down the bundle of His, and thence to the ventricular muscle. In these cases the stimuli arise in the auricle, which is for some reason in a state of irritability, and these waves from the fibrillating auricle are continually bombarding the bundle of His. As often as the conductivity of the bundle is sufficiently restored to allow of the passage of an impulse, one or the summation of several of these stimuli pass down the bundle and cause the ventricle to contract. The stimuli not being regular either in force or time will account for the irregularity of the ventricle. Whether the circulation can be carried on will depend on the sufficient filling of the ventricle through a possibly stenosed valve without the help of the auricular contraction, and on the power of the ventricle to continue contracting under such adverse circumstances. One of the chief factors in the maintaining of the flow of blood into the ventricles is probably the intra-auricular tension, which is high, and this in most cases serves to force the blood through the auriculo-ventricular valves and so to carry on the circulation.

With regard to the pathology of this condition, a fair number of autopsies with sections of the heart have been recorded. Unfortunately, attention has been paid to one part only of the heart by the various pathologists. Thus in Mackenzie's cases the bundle was the chief object examined; in other cases it has been the auricle. In most, however, fibrosis of the auricle has been found, and lesions in the bundle in a great many. Schonberg has recently examined five cases pretty thoroughly, and in all has discovered a chronic lymphocytic infiltration of the tissues at the junction of the superior vena cava and the auricle, that is just about the position of the normal pacemaker of the heart. Lewis suggests that fibrosis of the auricle, by interfering with its circulation, sets up a state of irritability of the auricular tissue, and that this causes firstly premature beats of the auricle and finally fibrillation.

The action of the digitalis group of drugs in these cases is very interesting. Most of those caused by rheumatism show a marked reaction, while many of those without a rheumatic history show no reaction at all. In the former the reaction is often very characteristic, but to obtain it the drug needs to be pushed—♏xx of the tincture three times a day is the usual dose. It is a noteworthy fact that vomiting and other signs of poisoning rarely set in before the heart reacts. The total amount necessary to cause reaction varies, but the average is from ʒiv to ℥i. As soon as the slowing begins diuresis sets in, œdema and dropsy clear up like magic, and very shortly the patient is perfectly comfortable. Mackenzie shrewdly suggests that the reputation of digitalis is based on its action in this class of case. Squills and strophanthus act in the same way, but require larger doses up to ʒii in the twenty-four hours, and they seem more apt to produce troublesome symptoms, such as diarrhœa.

If we analyse the result of digitalis upon the pulse, we find that there are three main types of reaction. (1) A simple slowing may take place, the pulse remaining completely irregular; (2) the pulse may be further slowed to 30 or 40 per minute, when it becomes quite regular, each beat being of the same size and length; and (3) slowing may take place with a persistent coupled rhythm, there being an alternation of a large beat followed by a small beat, and then a long pause before the next small beat. In this case the distance between the large beat and the small one is always the same, but the length of the long pause is quite irregular.

That digitalis and its allies have the power of slowing the pulse is well known. It may effect this in two ways

Fig. 3.

*Auricular fibrillation, showing simple slowing with digitalis* ♏ *x four times a day. The rate was* 112 *before the digitalis was given.*

at least. (1) In normally acting hearts, where the rhythmic stimuli start from the root of the great veins, that is from the sinus-node of Keith, it slows the heart by acting on the node through the vagus. Seeing that in cases of fibrillation the normal sinus rhythm is in abeyance, this method is not possible. (2) In cases where there is any defect in the bundle of His digitalis has been proved to further depress the conductivity of the bundle, and so variable degrees of heart block, where some or all stimuli fail to reach the ventricle, may be induced. In nearly all the rheumatic cases of auricular fibrillation some pathological condition of the bundle has been found, thereby allowing the inference to be drawn that some defect in conductivity existed during life. Digitalis, by increasing this defect, would cut off some of the irregular stimuli of the fibrillating auricle from the ventricle, and so a simple slowing of the pulse would occur, but it would still remain irregular. (Fig. 3.) If the digitalis causes a complete block, the ventricle, as in Stokes-Adams disease, may take on a rhythm of its own, and a regular beat of 30 to 40 to the minute occurs. (Fig. 4.) Owing to the better filling of the ventricle during the long diastole, the heart contracts more strongly, and so in some cases we may have the paradox of the patient being better with heart block than without it. The

**Fig. 4.**

*Auricular fibrillation, showing complete heart block under digitalis. Radial pulse is regular, beating 44 to the minute.*

auricles, as has been shown by electro cardiograms, continue to fibrillate as before.

The third variation (the coupled rhythm) has not, so far as I know, been satisfactorily explained up to the present. The second small beat has been proved to be a ventricular extra systole, that is a premature beat arising in the ventricle independently of the auricles. When either of these reactions has been obtained the digitalis may be lessened, as pushing it further will do no good and may do harm. A point I should like to accentuate is, that when a reaction to digitalis has been obtained the drug should not be stopped, but continued in slightly smaller doses. Too often digitalis has been cut off simply because the pulse has been slowed, and if this be done the rate invariably rises again. The heart must be kept slow, and one often finds that it can be maintained at any given pace according to the amount of the drug exhibited. As to the most desirable rate to be maintained, the patient's feelings are usually the best guide, and an intelligent patient can often regulate his own dose. I know one lady with auricular fibrillation who for years has taken digitalis according to her sensations, and has kept herself comparatively well. Another was in bed with dropsy, cyanosis and dyspnœa for eight months. She was given small doses of digitalis occasionally, but it seemed always to cause sickness. On giving her one of Nativelle's digitalin granules twice a day her pulse came down to 44 (see tracing, Fig. 4), and recently she walked over a quarter of a mile without discomfort. To stop the drug in these cases is to invite an attack of heart failure. Most of us remember patients who have come back again and again to hospital as soon as they gave up the out-patient department and their digitalis.

The treatment of cases that do not react to digitalis is unsatisfactory. When compensation has broken down they rarely regain it to any great extent, and the duration of their existence depends upon how long the ventricle can stand the rapid irregular contractions. Caffeine and nux vomica perhaps help as well as any drugs.

There is another rarer group of cases where the pulse is slow, usually about 50–60, but on analysis proves to be completely irregular, and the venous pulse is of the ventricular type. These are cases of auricular fibrillation, where there is an organic defect in conductivity either from arterial disease of the vessels supplying the bundle or from fibrosis of the bundle. These are the cases designated by Mackenzie in his book as nodal bradycardia. The condition may go a step farther, and a complete block in the bundle may be present, when the ventricle will take on its own rhythm of 30 per minute or thereabouts. In these cases, as in one published by Lewis and Mack, there may be epileptiform seizures, and indeed they only differ from the Stokes-Adams syndrome in having no co-ordinate contraction of the auricles.

The prognosis in these fibrillation cases depends largely on how the heart behaves under the new conditions. The inception of this abnormal rhythm causes in some patients sudden death immediately; in others acute dilatation of the heart with dropsy, dyspnœa, anuria, and enlargement of the liver; and again, other patients come to the physician without any idea that they have anything wrong with their hearts. Taking these seriatim, in the first prognosis is unnecessary. In the second case, with acute heart failure, the prognosis depends on the reaction to digitalis. If they react and go on taking the drug, they may live quite useful lives for years, provided they can take things quietly; if there be no reaction the prognosis is bad. In the cases that are unaware of their condition, although they get through the ordinary routine of daily life, one finds on questioning them that they are breathless on undertaking any unusual exertion, and that they are liable to discomfort or even pain in the region of the præcordium. In other words, the reserve power of their hearts is crippled, and the immediate prognosis depends on the amount of crippling. If it be slight, they have with care several years before the compensation breaks down, and when that occurs the future depends on their reaction to digitalis. In the cases with broken compensation which do not react to digitalis the prognosis is bad. They may live for some months if they lie in bed, but they slowly develop all the signs of chronic heart failure, and die in spite of all the drugs at our command.

Can auricular fibrillation be recognised without resort to polygraphic tracings? This is an important point, since most of us have little time and no machine. I think it can. If one relies on a continuous and complete irregularity, not an occasional one, a visible systolic pulsation in the veins of the neck, and signs of venous engorgement with the absence of a presystolic murmur, there will be few cases where the diagnosis will be wrong.

## Figures and Tables

**Fig. 1. f1:**
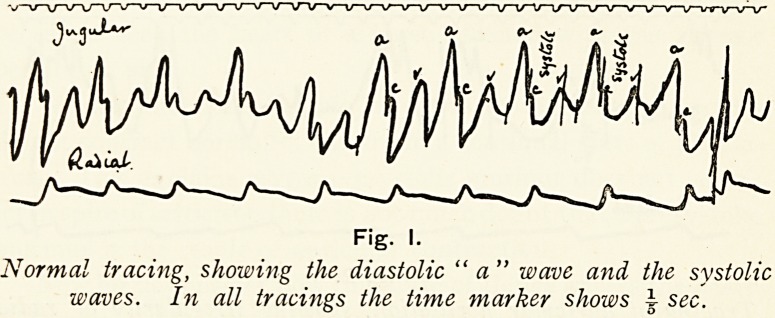


**Fig. 2. f2:**
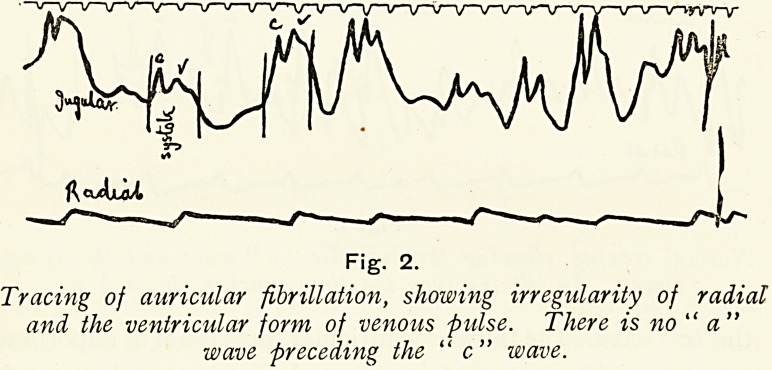


**Fig. 3 f3:**
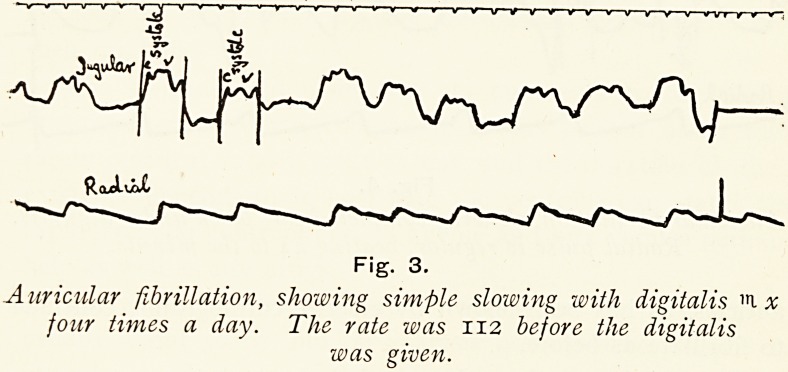


**Fig. 4. f4:**